# UV Light Induces Dedoping of Polyaniline

**DOI:** 10.3390/polym8020034

**Published:** 2016-01-28

**Authors:** Yuki Kaitsuka, Hiromasa Goto

**Affiliations:** Division of Materials Science, Faculty of Pure and Applied Sciences, University of Tsukuba, Tsukuba Ibaraki 305-8573, Japan; s-ykaitsuka@ims.tsukuba.ac.jp

**Keywords:** doping-dedoping, light induced dedoping, polyaniline

## Abstract

UV (Ultra-Violet) light-driven change in optical absorption of polyaniline (PANI) is reported. Irradiation of UV light to PANI/camphor sulfonic acid prepared by electrochemical polymerization allows dedoping of the PANI. Especially, UV light irradiation in the presence of a radical trap agent effectively reduces (dedoping) the PANI. The result in this study is quite simple; however, this may be a first report for light-induced dedoping (color change) of a conductive polymer.

## 1. Introduction

Polyaniline (PANI) is one of the most intriguing conjugated polymers because of the simple and convenient method required to synthesize it, and its tunable conductivity upon doping [1,2,3]. In the present study, we tune optical absorption by the irradiation of Ultra-Violet (UV) light. The irradiation of UV light induces dedoping of PANI. Radical trap reagent aids the dedoping. This phenomenon can be referred to as a new type photochromism.

## 2. Materials and Techniques

Aniline (Wako, Osaka, Japan) was purified by distillation prior to use. The (−)-Camphor sulfonic acid (Tokyo Chemical Industry (TCI), Tokyo, Japan), sulfuric acid (TCI) and ammonium peroxodisulfate (APS, Kanto Chemical, Tokyo, Japan) were used as received.

Ultra violet and visible (UV-vis) spectroscopy was recorded on a V-630 spectrophotometer (JASCO, Tokyo, Japan). UV light (λ = 375 nm, 0.3 mW/cm^2^) was irradiated to the PANI. Scanning electron microscope (SEM) observations were carried out with a JSM-7000F (JEOL, Tokyo, Japan) and JED-2200 (JEOL, Tokyo, Japan).

## 3. Synthesis

### Polyaniline doped with (−)-camphor sulfonic acid (PANI/(−)-CSA)

**Scheme 1:**
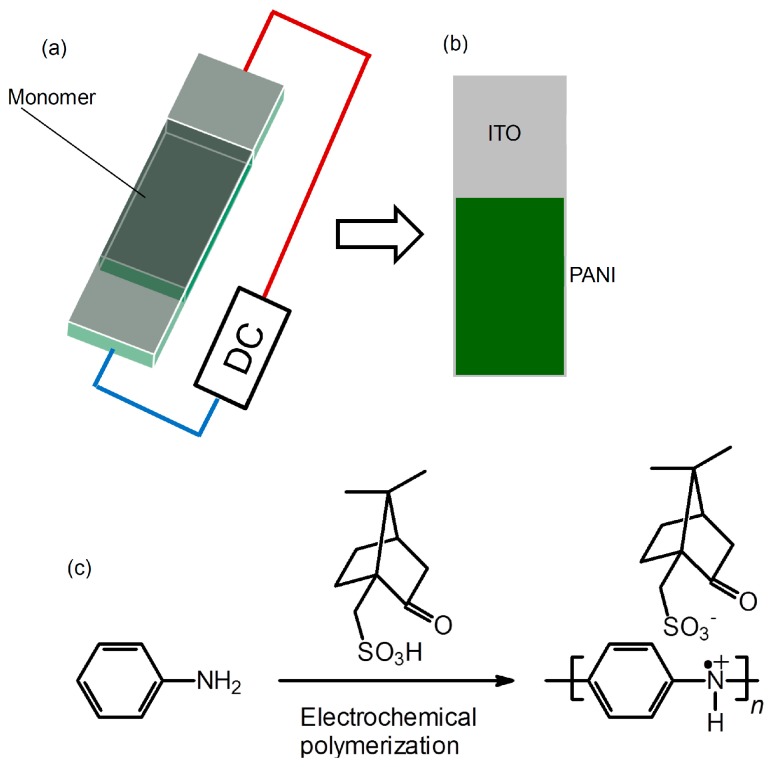
Preparation of PANI/(−)-CSA. (**a**) Electrochemical polymerization with the two-electrode method; (**b**) Resultant PANI on ITO; (**c**) Chemical formula for PANI.

PANI films on indium-tin-oxide (ITO)-coated glass were prepared with the electrochemical polymerization method. An electrolyte was prepared by addition of the aniline (20 mg), (−)-Camphor sulfonic acid (CSA) distilled water (1 mL). Before polymerization, aniline-CSA salt is formed. Then, polymerization was performed in a cell consisting of a sandwiched ITO electrode (cell gap = 0.2 mm) by applying a constant direct-current (DC) voltage of 3.0 V (Scheme 1a). This method we developed is referred to as sandwich cell polymerization [4]. The resultant film was deposited on the anode side of an indium-tin-oxide (ITO)-coated glass electrode (Scheme 1b). The film was washed with distilled water and methanol to remove low-molecular-weight fractions. The chemical formula of this reaction for PANI bearing CSA via static bond (PANI/(−)-CSA) thus prepared is shown in Scheme 1c.

## 4. Optical Spectroscopy

**Figure 1:**
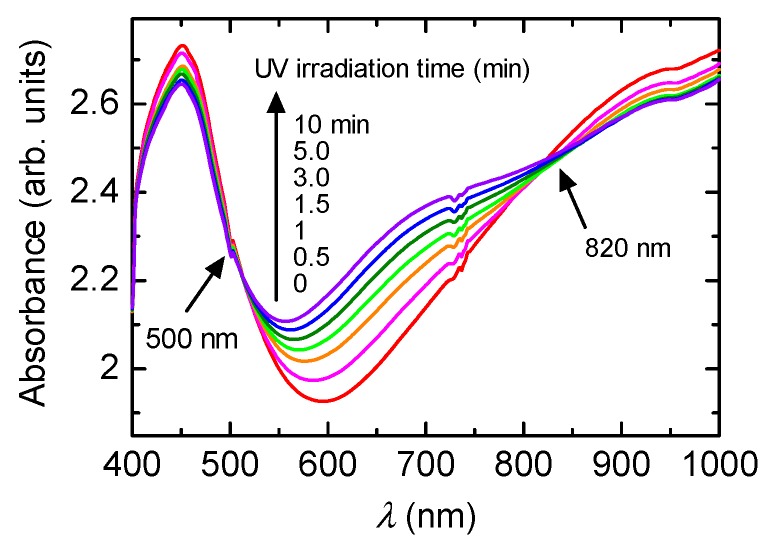
UV-vis absorption spectra of PANI/(−)-CSA under UV light irradiation.

Figure 1 shows the change in optical absorption with the irradiation of UV light. Absorptions at long wavelengths (Vis-NIR range) have doping bands (polarons and bipolarons). The polymer before irradiation of UV light is doped with (−)-CSA as a substituent. Sequential irradiation of UV light induces dedoping, resulting in the long wavelength absorption band being decreased and the absorption band at around 700 nm being increased. Isosbestic points at 500 and 800 nm are observed. This result indicates that the PANI has plural phases, such as emeraldine salt (before irradiation of the UV) and emeraldine base (after irradiation of the UV). 

**Figure 2:**
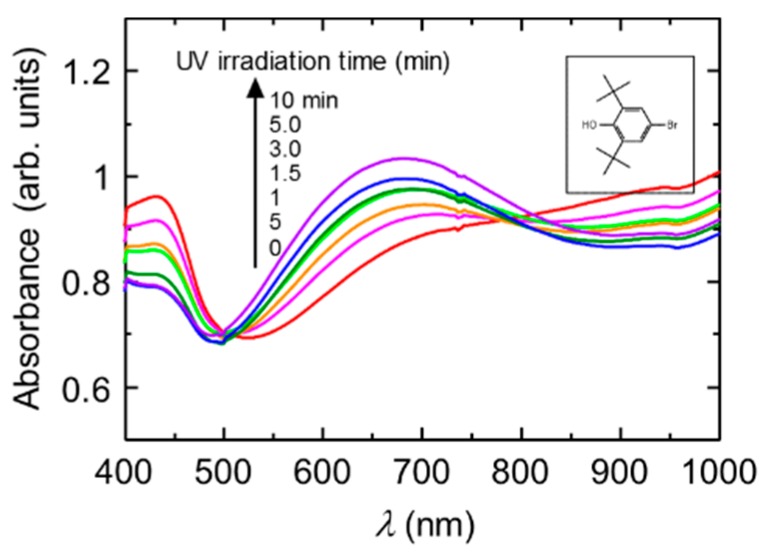
UV-vis absorption spectra of the PANI (0.01 M dispersion in the water) in the presence of 4-bromo-2,6-di-*tert*butyl phenol as a radical trap regent under UV light irradiation. Inset shows chemical structure of 4-bromo-2,6-di-*tert*butyl phenol.

Figure 2 shows the change in the optical absorption of the PANI in the presence of a radical trap reagent (0.01 M dispersion in water) with irradiation of UV light. The Figure 2 (inset) shows the chemical structure of 4-bromo-2,6-di-*tert*butyl phenol. Before irradiation of UV light, the PANI shows a doping band in the Vis-NIR range. On the other hand, irradiation of UV light changes the spectral form. The new absorption band was observed at 820 nm. This is due to the formation of the emeraldine base state of the main chain. Thus, light-induced dedoping from emeraldine salt to emeraldine base remarkably occurs. At the present stage, the mechanism of dedoping of the CSA-doped polyaniline is unclear. However, the radical trap agent drives the dedoping of the PANI under UV light.

## 5. Morphology

**Figure 3:**
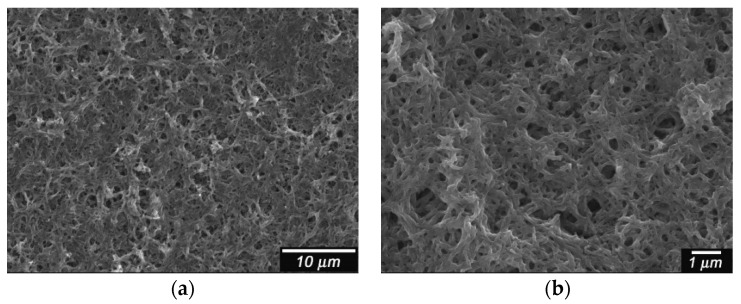
Scanning electron microscope (SEM) images of PANI/(−)-CSA. CSA = camphor sulfonic acid. (**a**) Low magnification; (**b**) High magnification.

Scanning electron microscope (SEM) images for the film are shown in Figure 3. PANI doped with CSA shows a network structure [5]. The magnification image shows that the electrochemical polymerization produces the anastomosis fibril structure of the PANI.

## 6. Conclusions

PANI/(−)-CSA shows photo-induced dedoping at the molecular level. This reaction is irreversible. A radical trap agent accelerates the dedoping. This result demonstrates that the radical trap agent provides electrons to the doped PANI with the aid of UV light irradiation. Although photo-induced doping and photo-conduction are well known phenomena, UV-induced dedoping (color change) is a first example in the research field of conducting polymers.
